# Social Distancing During the COVID-19 Pandemic and Neonatal Mortality in the US

**DOI:** 10.1001/jamanetworkopen.2024.22995

**Published:** 2024-07-18

**Authors:** Vivek V. Shukla, Lucinda J. Weaver, Avinash Singh, A. K. M. Fazlur Rahman, Arie Nakhmani, Colm P. Travers, Rachel Sinkey, Nitin Arora, Namasivayam Ambalavanan, Waldemar A. Carlo

**Affiliations:** 1 Division of Neonatology, Department of Pediatrics, University of Alabama at Birmingham; 2 Department of Electrical and Computer Engineering, University of Alabama at Birmingham; 3 Department of Biostatistics, University of Alabama at Birmingham; 4 Division of Maternal and Fetal Medicine, Department of Obstetrics and Gynecology, University of Alabama at Birmingham

## Abstract

**Importance:**

Neonatal mortality is a major public health concern that was potentially impacted by the COVID-19 pandemic. To prepare for future health crises, it is important to investigate whether COVID-19 pandemic–related interventions were associated with changes in neonatal mortality.

**Objective:**

To investigate whether social distancing during the pandemic was associated with a higher neonatal mortality rate.

**Design, Setting, and Participants:**

This cohort study examined maternal-linked birth and infant death records from the National Center for Health Statistics, a population-level US database, from 2016 through 2020. The mortality rates were correlated using machine learning–based autoregressive integrated moving average (ARIMA) models with the social distancing index (SDI). The reference period was January 2016 through February 2020, and the pandemic period was March through December 2020. Statistical analysis was performed from March 2023 to May 2024.

**Exposures:**

SDI, computed from 6 mobility metrics.

**Main Outcomes and Measures:**

The primary outcome was neonatal mortality rate, defined as death at age less than 28 days.

**Results:**

The study included 18 011 173 births, of which 15 136 596 were from the reference period (7 753 555 [51.22%] male; 11 643 094 [76.92%] with maternal age of 20 to 34 years) and 2 874 577 were from the pandemic period (1 472 539 [51.23%] male; 2 190 158 [76.19%] with maternal age of 20 to 34 years). Through ARIMA-adjusted analyses, accounting for the declining mortality trend in the reference period, the mortality rates during the pandemic period did not significantly differ from the expected rates. SDI did not exhibit significant correlations with neonatal mortality (unadjusted: correlation coefficient [CC], 0.14 [95% CI, −0.53 to 0.70]; ARIMA adjusted: CC, 0.29 [95% CI, −0.41 to 0.77]), early neonatal mortality (unadjusted: CC, 0.33 [95% CI, −0.37 to 0.79]; ARIMA adjusted: CC, 0.45 [95% CI, −0.24 to 0.84]), and infant mortality (unadjusted: CC, −0.09 [95% CI, −0.68 to 0.57]; ARIMA adjusted: CC, 0.35 [95% CI, −0.35 to 0.80]). However, lag analyses found that SDI was associated with higher neonatal and early neonatal mortality rates with a 2-month lag period, but not with infant mortality rate. SDI was also associated with increases in 22-to-27 weeks’ and 28-to-32 weeks’ preterm delivery with a 1-month lag period.

**Conclusions and Relevance:**

In this population-level study of National Center for Health Statistics databases, neonatal, early neonatal, and infant mortality rates did not increase during the initial COVID-19 pandemic period. However, associations were observed between the pandemic period social distancing measures and higher rates of neonatal and early neonatal mortality, as well as preterm birth rate with a lag period, suggesting the importance of monitoring infant health outcomes following pandemic-related population behavior changes.

## Key Points

**Question:**

Was more social distancing during the COVID-19 pandemic associated with neonatal mortality?

**Findings:**

In this population-level cohort study of 18 011 173 births from 2016 to 2020, increased social distancing was not associated with higher neonatal mortality. In an analysis using a 2-month lag period, an association was observed.

**Meaning:**

This study’s results suggest the importance of monitoring infant health outcomes following pandemic-related population behavior changes.

## Teaser

This cohort study investigates whether social distancing during the COVID-19 pandemic was associated with neonatal mortality rates in the US.

## Introduction

The COVID-19 pandemic affected the health care system globally,^1,2^ resulting in more than 1.1 million deaths in the United States and close to 7 million deaths worldwide.^3^ The virus was declared a public health emergency of international concern by the World Health Organization on January 30, 2020.^4^ This was followed by many countries declaring national public health emergencies and public health interventions such as regional and national lockdowns, school and workplace closures, restrictions on public gatherings, stay-at-home restrictions, travel restrictions, social distancing, and masking requirements.^5^

Studies show that social distancing and other public health measures effectively reduced the spread of COVID-19 and other infections.^6,7^ However, they also had unanticipated effects, such as reduced health care accessibility and utilization,^8,9^ even for potentially serious illnesses,^10^ and increased morbidity and mortality.^11^ Pregnant people also encountered substantial obstacles in accessing health care during the pandemic period,^12,13^ that in addition to the infection itself may have contributed toward increased maternal and fetal mortality and pregnancy-related morbidities identified in the previous publications.^14,15^ It is conceivable that neonatal and infant outcomes during the pandemic period were also affected.

However, population-based studies on perinatal, neonatal, and infant outcomes in relation to the pandemic have had varied findings.^15,16^ There is a lack of national population-based studies correlating objective pandemic-related social distancing and mobility metrics and infant outcomes. We published a population-based study from Alabama that assessed the social distancing index (SDI) and fetal and neonatal outcomes where we found that SDI was not significantly correlated with the percentage change of stillbirths and neonatal mortality^17^; however, as it was based on data from only 1 state, the generalizability of our previous study could be limited. Regional variability associated with the pandemic related to the implementation, public adherence to lockdowns, and mobility restrictions provided an opportunity to objectively examine the changes in infant outcomes with pandemic-related social distancing metrics. Analyzing the changes in the epidemiology of adverse neonatal and infant outcomes with an objective SDI may help inform health policy and response preparedness for the present pandemic and future health care disruptions.

Maternal pregnancy complications increase risk of preterm delivery and neonatal morbidity and are a major contributor toward neonatal mortality.^18^ Consequently, it is plausible that the COVID-19 pandemic mobility restrictions and reduced access to antenatal and perinatal care may have been associated with increased neonatal mortality. Therefore, we hypothesized that increased social distancing during the pandemic was associated with a higher neonatal mortality rate.

## Methods

This cohort study was approved by the University of Alabama at Birmingham institutional review board, and a waiver of informed consent was granted as this was an observational database-based study. We followed the Strengthening the Reporting of Observational Studies in Epidemiology (STROBE) reporting guideline.^19^

This study used a population-based cohort design, utilizing data from the National Center for Health Statistics (NCHS) of the Centers for Disease Control and Prevention (CDC) maternal-linked birth and infant death records spanning from January 2016 through December 2020. Participants included all live births, excluding infants with less than or equal to 21 weeks’ gestational age or greater than or equal to 42 weeks’ gestational age at birth to avoid extreme outliers, categorized into 2 periods: (1) the reference period (January 2016 through February 2020) and (2) the pandemic period (March through December 2020). Neonatal mortality was defined as death at less than 28 days, early neonatal mortality was defined as death at less than 7 days, and infant mortality was defined as death at less than 365 days. The primary outcome was neonatal mortality rate, and the secondary outcomes were early neonatal and infant mortality rates, and the gestational age at delivery, categorized as 22 to 27 weeks (extremely preterm), 28 to 32 weeks (moderately preterm), 33 to 36 weeks (late preterm), and 37 to 41 weeks (term) deliveries. Rates were expressed per 1000 live births. Reference period rates were calculated as mean of monthly rates in the reference period. CDC definitions were used to define the variables.^18^

### Statistical Analysis

We used descriptive analysis (χ^2^, Mann-Whitney *U*, and Welch *t* tests, as applicable) to compare the demographic and clinical characteristics and outcomes between the study groups. We conducted interrupted time-series analysis using machine learning–based Box and Jenkins autoregressive integrated moving average (ARIMA) models. The autoregressive (AR), differencing (I), and moving average (MA) parts of these models work well to deal with stationarity, seasonality, and trends in outcome rates. This involved building models using the reference period data of 50 consecutive months from January 2016 to February 2020, and forecast outcome rates in the pandemic period consisting of 10 months from March 2020 to December 2020. To assess the potential association with the pandemic, we added a categorical variable called pandemic onset to the best-fitting ARIMA model and compared observed outcome rates to the expected rates. Outcomes outside of this range were classified as unusual. We evaluated the residuals of the final models to ensure the independence of monthly observations and the accuracy of the model output. Additionally, we also investigated the correlation between outcome rates with the SDI, defined as the extent to which residents of the state practiced social distancing, computed from 6 mobility metrics (including percentage staying home, percentage reduction of all trips compared with pre–COVID-19 benchmark, percentage reduction of work trips, percentage reduction of nonwork trips, percentage reduction of travel distance, and percentage reduction of out-of-county trips).^20^ To assess the association between outcomes and SDI, we calculated the percentage change in outcome rates in the pandemic compared with the reference period rates ([pandemic − reference] / reference × 100) and ARIMA-expected outcome rates ([pandemic − expected] / expected × 100). We used Pearson correlation coefficient to measure the strength and direction of the correlation between the percentage change of outcomes and SDI. We also conducted correlation analyses between outcomes and SDI using monthly lag periods to explore temporal dynamics. This approach aimed to understand how changes in SDI over time were associated with mortality rates and gestational week metrics, considering potential delayed effects of pandemic-related social distancing measures. These analyses were chosen based on previous findings associating COVID-19 cases with higher morbidity and mortality after a lag period.^21,22,23^

Using the independent sample test for dichotomous outcomes, a sample size of 15 136 596 from the reference period, and 2 874 577 from the pandemic period was sufficient to detect a 3 or more percentage difference in neonatal mortality rate between the reference and pandemic period, assuming a power of 0.80 and α of .05, and using neonatal mortality rate of 3.69 out of 1000 live births as reported by the CDC for 2019.^24^ Acknowledging the hierarchical nature of the data, with infants nested within hospitals, and hospitals possibly within cities and rural areas of varying sizes and academic settings, we accounted for intraclass correlations. Using a conservative design effect of 2 accommodated potential variations in intraclass correlations. Typically, in NCHS complex sample surveys, the design effect rarely exceeds 2.^25^ The design effect can be expressed as DE = 1 + (m − 1) × ICC, where DE is the design effect, m is the mean cluster size, and ICC is the intraclass correlation. Assuming a mean cluster size of 100 participants, the design effect of 2 supports an ICC of 0.01. Considering the design effect of 2, we determined that we needed 100 000 infants from each period to maintain at least 80% power to detect a 0.15% difference between periods. The available sample size was considered adequate to detect meaningful differences in neonatal mortality rates between the reference and pandemic periods.

The study used 2-tailed hypotheses and deemed *P* < .05 as a measure of statistical significance. Analyses were conducted from March 2023 to May 2024 using SPSS Statistics version 29 (IBM Corp), Python version 3.10 (Python Software Foundation), and SAS version 9.4 (SAS Institute).

## Results

A total of 18 999 835 live births were available in the database. After exclusions, the study included 18 011 173 births, of which 15 136 596 were from the reference period (7 753 555 [51.22%] male; 1 048 146 [6.92%] with Asian maternal race, 2 419 243 [15.98%] Black maternal race, 11 089 016 [73.26%] White maternal race, 580 191 [3.83%] other maternal race; 11 643 094 [76.92%] with maternal age of 20 to 34 years), and 2 874 577 were from the pandemic period (1 472 539 [51.23%] male; 185 054 [6.44%] with Asian maternal race, 464 050 [16.14%] Black maternal race, 2 107 232 [73.31%] White maternal race, 118 241 [4.11%] other maternal race; 2 190 158 [76.19%] with maternal age of 20 to 34 years) (eFigure 1 in Supplement 1). Comparisons of demographic variables between the study groups showed an increase in maternal age, education level, body mass index (calculated as weight in kilograms divided by height in meters squared), pregestational diabetes, and pregestational hypertension in the pandemic period. Among the prenatal variables, prenatal visits were lower in the pandemic period, and the rates of gestational diabetes, gestational hypertension, induction of labor, and neonatal intensive care unit admission were higher in the pandemic period. Extremely and moderately preterm births were lower during the pandemic period (extremely preterm births during the reference period: 0.63% [95 637 of 15 136 596] vs pandemic period: 0.60% [17 366 of 2 874 577]; moderately preterm births during the reference period: 1.90% [287 785 of 15 136 596] vs the pandemic period: 1.87% [53 777 of 2 874 577]; both *P* < .001), whereas late preterm births were higher in the pandemic period (reference period: 9.73% [1 473 068 of 15 136 596] vs pandemic period: 9.94% [285 706 of 2 874 577], *P* < .001). Neonatal, early neonatal, and infant mortality rates were lower during the pandemic period (neonatal mortality rate during the reference period: 2.93 per 1000 live births vs pandemic period: 2.73 per 1000 live births; early neonatal mortality rate during the reference period: 2.19 per 1000 live births vs pandemic period: 2.03 per 1000 live births; infant mortality rate during the reference period: 4.86 per 1000 live births vs pandemic period: 4.59 per 1000 live births; all *P* < .001) (Table 1). On ARIMA model analyses, all observed mortality and gestational age birth rates during the pandemic period were within the expected 95% CIs (Table 2 and Table 3; eFigures 2 and 3 in Supplement 1), suggesting that when adjusted for the trend in reference period there were no significant changes in outcomes during the pandemic period.

**Table 1.** Baseline Characteristics and Outcomes

Variables	Infants, No. (%)		*P* value
	Reference period (n = 15 136 596)	Pandemic period (n = 2 874 577)	
Maternal age, y			
≤19	739 345 (4.88)	123 766 (4.31)	<.001
20-34	11 643 094 (76.92)	2 190 158 (76.19)	
≥35	2 754 157 (18.20)	560 653 (19.50)	
Maternal race, No. (%)^a^			
Asian	1 048 146 (6.92)	185 054 (6.44)	.17
Black	2 419 243 (15.98)	464 050 (16.14)	
White	11 089 016 (73.26)	2 107 232 (73.31)	
Other	580 191 (3.83)	118 241 (4.11)	
Maternal education			
≤12th grade	1 924 193 (12.71)	329 148 (11.45)	<.001
High school	3 810 926 (25.18)	744 497 (25.90)	
Some college/associate’s degree	4 260 649 (28.15)	781 372 (27.18)	
Bachelor’s degree	3 094 418 (20.44)	608 281 (21.16)	
Master’s or higher	1 844 833 (12.19)	370 376 (12.88)	
Unknown	201 577 (1.33)	40 903 (1.42)	
Maternal BMI, mean (SD)	28.83 (13.03)	28.90 (11.96)	<.001
Pregestational diabetes	141 849 (0.94)	31 095 (1.08)	<.001
Pregestational hypertension	305 549 (2.02)	74 696 (2.60)	<.001
No. of prenatal visits, mean (SD)	13.54 (14.55)	12.97 (13.86)	<.001
Gestational diabetes	991 044 (6.55)	231 334 (8.05)	<.001
Gestational hypertension	1 058 964 (7.00)	245 681 (8.55)	<.001
Induction of labor	4 015 333 (26.53)	901 666 (31.37)	<.001
Maternal chorioamnionitis	235 676 (1.56)	44 680 (1.55)	.88
Cesarean delivery	4 856 148 (32.08)	921 651 (32.06)	.44
Maternal ICU admission, No. (No./100 000 live births)	24 405 (161.23)	4852 (168.79)	.41
Infant sex, No. (%)			
Male	7 753 555 (51.22)	1 472 539 (51.23)	.99
Female	7 383 041 (48.78)	1 402 038 (48.77)	
Birth weight, mean (SD), g	3257 (604)	3253 (594)	<.001
NICU admission	1 398 438 (9.24)	272 674 (9.49)	<.001
Gestational age, wk			
22-27	95 637 (0.63)	17 366 (0.60)	<.001
28-32	287 785 (1.90)	53 777 (1.87)	
33-36	1 473 068 (9.73)	285 706 (9.94)	
37-41	13 280 106 (87.74)	2 517 728 (87.59)	
Age at infant death, mean (SD), d	51.96 (78.65)	52.66 (78.95)	.33
Mortality outcomes, No. (No./1000 live births)			
Neonatal mortality	44 382 (2.93)	7849 (2.73)	<.001
Early neonatal mortality	33 148 (2.19)	5838 (2.03)	<.001

Abbreviations: BMI, body mass index (calculated as weight in kilograms divided by height in meters squared); ICU, intensive care unit; NICU, neonatal intensive care unit.

^a^ Maternal race was categorized based on self-reported race. The other maternal race subcategory included American Indian and Alaska Native, Native Hawaiian and other Pacific Islanders, and more than 1 race.

**Table 2.** Observed and ARIMA-Expected Neonatal, Early Neonatal, and Infant Mortality Rates in the Pandemic Period^a^

Death month	Neonatal mortality			Early neonatal mortality			Infant mortality		
	Reference	Observed	Expected (95% CI)	Reference	Observed	Expected (95% CI)	Reference	Observed	Expected (95% CI)
March	2.92	2.56	2.78 (2.55-3.02)	2.17	1.96	2.05 (1.84-2.26)	5.02	4.51	4.68 (4.32-5.04)
April	3.10	2.89	2.77 (2.53-3.01)	2.36	2.23	2.04 (1.83-2.25)	5.04	4.63	4.62 (4.22-5.01)
May	2.95	2.77	2.76 (2.52-3.00)	2.19	2.10	2.03 (1.82-2.24)	4.89	4.66	4.59 (4.19-4.98)
June	3.06	2.93	2.75 (2.51-2.99)	2.31	2.21	2.02 (1.81-2.23)	4.97	4.64	4.57 (4.17-4.97)
July	2.89	2.75	2.74 (2.50-2.97)	2.17	2.02	2.01 (1.80-2.22)	4.61	4.37	4.56 (4.16-4.96)
August	2.86	2.64	2.72 (2.49-2.96)	2.13	1.95	2.00 (1.79-2.21)	4.57	4.39	4.55 (4.15-4.95)
September	2.87	2.64	2.71 (2.47-2.95)	2.14	1.87	1.99 (1.79-2.20)	4.70	4.47	4.54 (4.14-4.94)
October	2.91	2.62	2.70 (2.46-2.94)	2.14	2.01	1.99 (1.78-2.20)	4.81	4.50	4.54 (4.14-4.94)
November	2.89	2.71	2.69 (2.45-2.93)	2.13	1.95	1.98 (1.77-2.19)	4.83	4.72	4.53 (4.13-4.93)
December	2.88	2.72	2.68 (2.44-2.92)	2.10	1.98	1.97 (1.76-2.18)	4.97	4.59	4.52 (4.12-4.92)

Abbreviation: ARIMA, Box and Jenkins autoregressive integrated moving average.

^a^ Rates per 1000 live births.

**Table 3.** Observed and ARIMA-Expected Births at Gestational Age of 22-27 Weeks, 28-32 Weeks, 33-36 Weeks, and 37-41 Weeks in the Pandemic Period^a^

Birth month	Gestational age, wk											
	22-27			28-32			33-36			37-41		
	Reference	Observed	Expected (95% CI)	Reference	Observed	Expected (95% CI)	Reference	Observed	Expected (95% CI)	Reference	Observed	Expected (95% CI)
March	6.51	6.21	6.14 (5.64-6,64)	19.45	18.57	19.27 (17.74-20.80)	97.78	98.47	101.21 (94.95-107.47)	877.82	876.75	873.47 (866.23-880.70)
April	6.80	6.41	6.10 (5.44-6.75)	19.77	19.49	19.28 (17.37-21.18)	96.27	96.07	100.77 (94.06-107.48)	878.34	878.03	874.44 (865.64-883.25)
May	6.68	6.43	6.04 (5.31-6.77)	20.14	19.93	18.98 (16.77-21.19)	98.18	101.22	100.48 (93.67-107.29)	876.12	872.42	875.03 (865.47-884.60)
June	6.65	6.36	6.00 (5.24-6.75)	19.81	19.47	18.75 (16.49-21.01)	99.04	102.89	100.23 (93.38-107.08)	876.18	871.28	875.11 (865.35-884.86)
July	6.19	5.89	5.96 (5.20-6.72)	19.56	19.69	18.71 (16.45-20.96)	99.46	102.44	99.99 (93.12-106.87)	876.06	871.98	875.27 (865.51-885.02)
August	5.95	5.84	5.92 (5.16-6.69)	17.40	17.33	18.66 (16.40-20.92)	95.87	99.53	99.77 (92.87-106.67)	882.21	877.30	875.43 (865.68-885.18)
September	5.94	5.57	5.89 (5.12-6.66)	17.65	17.98	18.61 (16.36-20.87)	89.83	92.86	99.54 (92.62-106.46)	887.25	883.59	875.59 (865.84-885.35)
October	6.20	5.85	5.86 (5.09-6.62)	17.82	17.88	18.57 (16.31-20.83)	97.04	102.80	99.31 (92.37-106.26)	879.78	873.47	875.75 (866.00-885.51)
November	6.18	5.78	5.82 (5.06-6.59)	18.73	17.68	18.52 (16.26-20.78)	95.98	98.44	99.09 (92.12-106.05)	880.63	878.09	875.91 (866.16-885.67)
December	6.27	6.11	5.79 (5.02-6.56)	19.39	19.08	18.48 (16.22-20.74)	100.38	98.93	98.86 (91.88-105.85)	875.10	875.88	876.08 (866.32-885.83)

Abbreviation: ARIMA, Box and Jenkins autoregressive integrated moving average.

^a^ Rates per 1000 live births.

The SDI was not significantly correlated with neonatal mortality (unadjusted analysis: correlation coefficient [CC], 0.14 [95% CI, −0.53 to 0.70]; ARIMA-adjusted analysis: CC, 0.29 [95% CI, −0.41 to 0.77]), early neonatal mortality (unadjusted: CC, 0.33 [95% CI, −0.37 to 0.79]; ARIMA adjusted: CC, 0.45 [95% CI, −0.24 to 0.84]), and infant mortality (unadjusted: CC, −0.09 [95% CI, −0.68 to 0.57] and ARIMA adjusted: CC, 0.35 [95% CI, −0.35 to 0.80]). The SDI was also not significantly correlated with births at 22 to 27 weeks (unadjusted: CC, 0.04 [95% CI, −0.60 to 0.65]; ARIMA adjusted: CC, 0.45 [95% CI, −0.24 to 0.84]), 28 to 32 weeks (unadjusted: CC, −0.07 [95% CI, −0.67 to 0.58]; ARIMA adjusted: CC, 0.11 [95% CI, −0.55 to 0.69]), 33 to 36 weeks (unadjusted: CC, −0.25 [95% CI, −0.76 to 0.45]; ARIMA adjusted: CC, −0.49 [95% CI, −0.85 to 0.19]), and 37 to 41 weeks (unadjusted: CC, 0.35 [95% CI, −0.35 to 0.80]; ARIMA adjusted: CC, 0.31 [95% CI, -0.39 to 0.78]) (Figure).

**Figure.**
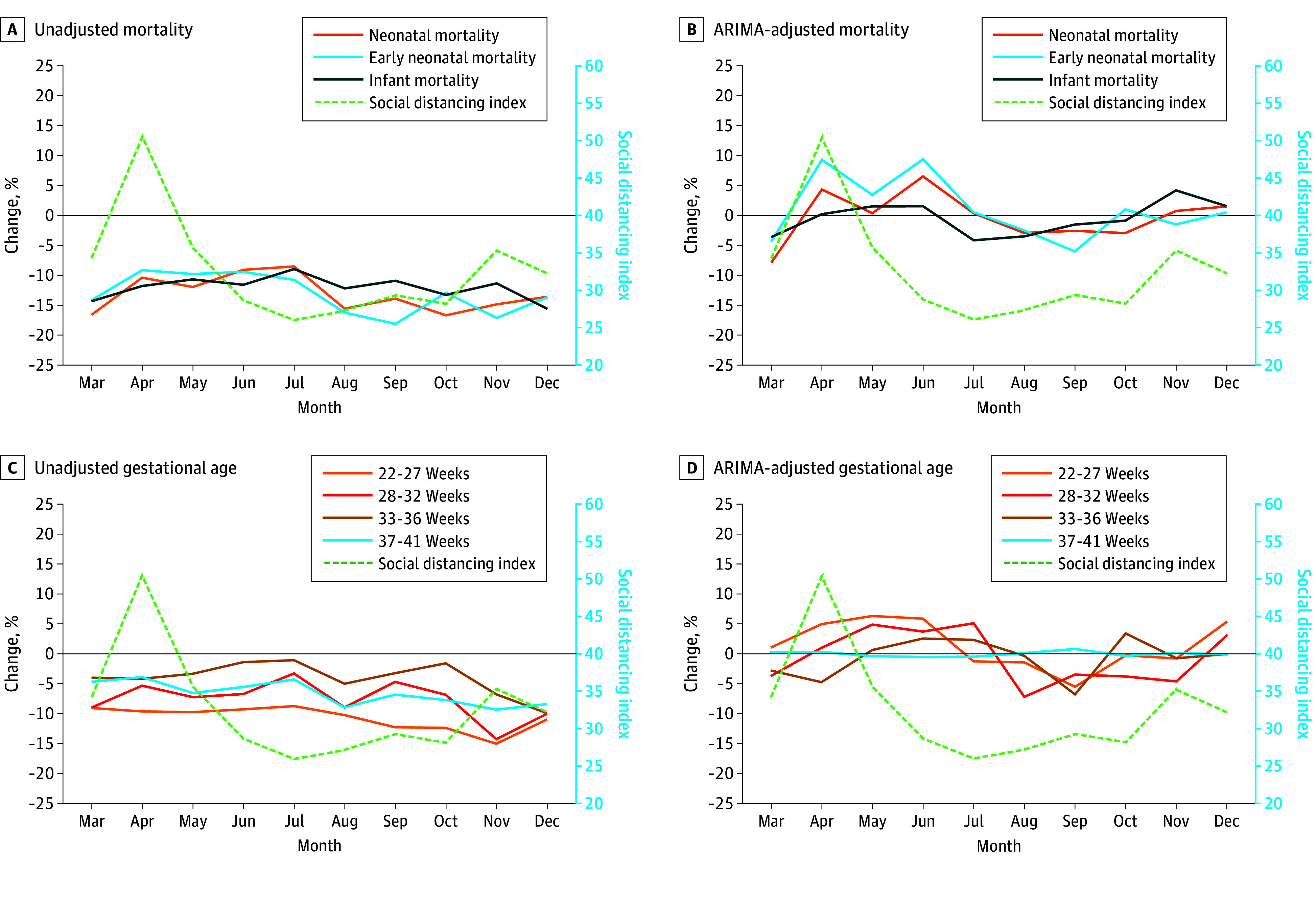
Social Distancing Index and Percentage Change in Neonatal Mortality, Early Neonatal Mortality, Infant Mortality, and Birth Gestational Age The percentage change in outcome rates during the pandemic compared with the reference period rates ([pandemic − reference] / reference × 100) and Box and Jenkins autoregressive integrated moving average (ARIMA) expected outcome rates ([pandemic − expected] / expected × 100) was calculated using the monthly rates in the pandemic period and corresponding reference period or ARIMA expected rates, for unadjusted (panels A and C) and ARIMA-adjusted comparison (panels B and D), respectively. Month-corresponding social distancing index was used for correlation.

SDI and mortality correlation with lag analyses showed that the SDI was significantly associated with higher neonatal mortality rates with a 2-month lag period (unadjusted: CC, 0.78 [95% CI, 0.17 to 0.95]; ARIMA adjusted: CC, 0.87 [95% CI, 0.42 to 0.97]), and with higher early neonatal mortality rates also with a 2-month lag period (adjusted: CC, 0.72 [95% CI, 0.04 to 0.94]; ARIMA adjusted: CC, 0.87 [95% CI, 0.44 to 0.97]) (Table 4). SDI was also significantly associated with higher birth rates during 22 to 27 weeks and 28 to 32 weeks with a 1-month lag period (22 to 27 weeks, ARIMA adjusted: CC, 0.78 [95% CI, 0.24 to 0.95]; 28 to 32 weeks, ARIMA adjusted: CC, 0.66 [95% CI, 0.01 to 0.92]) (eTable in Supplement 1).

**Table 4.** Lag Period Unadjusted vs ARIMA-Adjusted Analysis of Correlation of Social Distancing Index With Neonatal, Early Neonatal, and Infant Mortality

Lag period	CC (95% CI)					
	Neonatal mortality		Early neonatal mortality		Infant mortality	
	Unadjusted	ARIMA adjusted	Unadjusted	ARIMA adjusted	Unadjusted	ARIMA adjusted
Months						
0	0.14 (−0.53 to 0.70)	0.29 (−0.41 to 0.77)	0.33 (−0.37 to 0.79)	0.45 (−0.24 to 0.84)	−0.09 (−0.68 to 0.57)	0.35 (−0.35 to 0.80)
1	0.32 (−0.43 to 0.81)	0.38 (−0.38 to 0.83)	0.62 (−0.07 to 0.91)	0.49 (−0.24 to 0.87)	−0.002 (−0.66 to 0.66)	0.42 (−0.33 to 0.84)
2	0.78 (0.17 to 0.95)	0.87 (0.42 to 0.97)	0.72 (0.04 to 0.94)	0.87 (0.44 to 0.97)	0.30 (−0.51 to 0.83)	0.15 (−0.61 to 0.77)
3	0.74 (−0.02 to 0.95)	0.16 (−0.67 to 0.81)	0.50 (−0.40 to 0.91)	0.16 (−0.67 to 0.81)	0.63 (−0.22 to 0.94)	−0.63 (−0.94 to 0.22)

Abbreviation: ARIMA, Box and Jenkins autoregressive integrated moving average.

## Discussion

The current study aimed to test the hypothesis that increased social distancing during the pandemic was associated with a higher neonatal mortality rate in the United States using the population-level National Center for Health Statistics maternal linked-birth and infant death database from 2016 to 2020. The results showed no significant correlation between the SDI and neonatal mortality, early neonatal mortality, and infant mortality rates, and births across gestational age categories. However, statistically significant associations between the SDI and higher neonatal and early neonatal mortality with a 2-month lag period, and higher births at 22 to 27 weeks’ and 28 to 32 weeks’ gestational age with a 1-month lag period were found. Infant mortality rates were not found to be significantly associated with the SDI. These findings highlight the importance of monitoring the unintended consequences of pandemic-related population behavior changes on access to care among at-risk groups such as pregnant people and infants. Studies have shown that social distancing and other public health measures were effective in reducing the spread of COVID-19 and other infections,^6,7^ and reduced COVID-19 mortality.^26,27^ However, its impact on other diseases, particularly in at-risk groups is underexplored. There have been several studies that have assessed the changes in perinatal, neonatal, and infant mortality in relation to the COVID-19 pandemic and have shown varied findings.^15,16^ Meta-analyses of the published studies have shown that preterm delivery at less than 37 weeks (population-based studies from high-income countries: odds ratio [OR], 0.99 [95% CI, 0.97-1.01]) and neonatal mortality (OR, 1.17 [95% CI, 0.81-1.70]) in the pandemic period were not significantly different from the reference period.^16^

Similar to the current study finding of a statistically significant decrease in prenatal care visits, several studies have reported decreases and disruptions in prenatal care during the pandemic period.^28,29,30,31^ The finding of significant associations between the SDI and higher neonatal and early neonatal mortality with a 2-month lag period, and higher births at 22 to 27 weeks’ and 28 to 32 weeks’ gestational age with a 1-month lag period could possibly be due to disruptions in prenatal care and the resultant pregnancy complications that were more pronounced not immediately but after a lag period. Similar associations of COVID-19 cases and higher morbidity and mortality with a lag period have been reported,^21,22,23^ however, the current study is the first national population-based study in our knowledge to correlate objective pandemic-related social distancing and mobility metrics with infant outcomes with lag period analyses. This study is more generalizable than our previous study correlating SDI and perinatal outcomes,^17^ as this study includes all US states. While our previous study did not find a significant correlation between the SDI and neonatal mortality without a lag period, the current study, benefiting from a larger sample size and access to births from all US states, also conducted lag period analyses of the SDI, which revealed a novel association between the SDI and higher neonatal mortality and preterm birth with a lag period.

### Limitations

The limitations of this study include its observational design limiting the correlation we found to an association, which does not establish causality; we acknowledge this limitation but recognize that the pandemic created a natural experiment where access to prenatal care was disrupted. The study findings are subject to residual unaccounted confounding given the regional variations in pandemic-related population health interventions and the pandemic severity; however, the large sample size and rigorous statistical methods, including machine learning–based ARIMA-adjusted analyses, to account for temporal trends, increase the reliability of the findings. The SDI was measured at the state level, and specific practices may have varied within states; however, the use of this index provides a standardized measure for comparison of social distancing across multiple states, enhancing the validity of the study findings. The study’s focus on the association between social distancing and neonatal mortality rates provides valuable insights, although the effects of other pandemic-related factors were not analyzed, and future research is needed to address them.

## Conclusions

This cohort study highlights the potential unintended adverse effects of pandemic-related population behavior changes on infant health. Although no significant correlation was found between the SDI and neonatal mortality, early neonatal mortality, infant mortality rates, and births across gestational age categories, statistically significant associations were observed between the SDI and higher neonatal and early neonatal mortality rates, and births at 22 to 27 weeks’ and 28 to 32 weeks’ gestational age with lag period. These findings underscore the importance of considering the broader implications of pandemic-related population behavior changes and the need for in-depth concurrent analyses of their impact among at-risk populations. Further research is needed to examine the potential interactions of SDI with other objective pandemic-related indices that may affect infant mortality. This study also underscores the need for continued research to guide evidence-based public health policy for the current and future pandemics.

## Notes

Supplement 1. **eTable.** Lag Period Unadjusted vs. ARIMA Adjusted Analysis of Correlation of Social Distancing Index with Birth Gestational Age **eFigure 1.** Participant Flow Diagram **eFigure 2.** Study Period Observed and ARIMA Expected Pandemic Period Neonatal, Early Neonatal, and Infant Mortality Rates **eFigure 3.** Study Period Observed and ARIMA Expected Pandemic Period Births at Gestational Age of 22-27 Weeks, 28-32 Weeks, 33-36 Weeks, and 37-41 Weeks

Supplement 2.
Data Sharing Statement
