# Supplementary material for: Social Distancing During the COVID-19 Pandemic and Neonatal Mortality in the US
Source: JAMA Netw Open. 2024 Jul 18;7(7):e2422995. doi: 10.1001/jamanetworkopen.2024.22995 (PMC11258585; doi:10.1001/jamanetworkopen.2024.22995)
Supplement: Supplement 1. — eTable. Lag Period Unadjusted vs. ARIMA Adjusted Analysis of Correlation of Social Distancing Index with Birth Gestational Age eFigure 1. Participant Flow Diagram eFigure 2. Study Period Observed and ARIMA Expected Pandemic Period Neonatal, Early Neonatal, and Infant Mortality Rates eFigure 3. Study Period Observed and ARIMA Expected Pandemic Period Births at Gestational Age of 22-27 Weeks, 28-32 Weeks, 33-36 Weeks, and 37-41 Weeks [file jamanetwopen-e2422995-s001.pdf]

Supplemental Online Content

Shukla VV, Weaver LJ, Singh A, et al. Social distancing during the COVID-19 pandemic and neonatal mortality in the US. *JAMA Netw Open.* 2024;7(7):e2422995. doi:10.1001/jamanetworkopen.2024.22995

eTable 1. Lag Period Unadjusted vs. ARIMA Adjusted Analysis of Correlation of Social Distancing Index with Birth Gestational Age

eFigure 1. Participant Flow Diagram

eFigure 2. Study Period Observed and ARIMA Expected Pandemic Period Neonatal, Early Neonatal, and Infant Mortality Rates

eFigure 3. Study Period Observed and ARIMA Expected Pandemic Period Births at Gestational Age of 22-27 Weeks, 28-32 Weeks, 33-36 Weeks, and 37-41 Weeks.

This supplemental material has been provided by the authors to give readers additional information about their work.

© 2024 Shukla VV. *JAMA Network Open.*

eTable 1: Lag Period Unadjusted vs. ARIMA Adjusted Analysis of Correlation of Social Distancing Index with Birth Gestational Age

Lag Period	Gestational age							
	22-27 Weeks		28-32 Weeks		33-36 Weeks		37 - 41 Weeks	
	Unadjusted	ARIMA adjusted	Unadjusted	ARIMA adjusted	Unadjusted	ARIMA adjusted	Unadjusted	ARIMA adjusted
**Months**	**CC (95% CI)**	**CC (95% CI)**	**CC (95% CI)**	**CC (95% CI)**	**CC (95% CI)**	**CC (95% CI)**	**CC 95% CI**	**CC 95% CI**
**0**	0.04 (-0.60, 0.65)	0.45 (-0.24, 0.84)	-0.07 (-0.67, 0.58)	0.11 (-0.55, 0.69)	-0.25 (-0.76, 0.45)	-0.49 (-0.85, 0.19)	0.35 (-0.35, 0.80)	0.31 (-0.39, 0.78)
**1**	0.37 (-0.38, 0.83)	**0.78 (0.24, 0.95)**	0.04 (-0.63, 0.69)	**0.66 (0.005, 0.92)**	0 (-0.66, 0.66)	0.16 (-0.56, 0.74)	0.23 (-0.51, 0.77)	-0.41 (-0.84, 0.34)
**2**	0.56 (-0.23, 0.90)	0.55 (-0.24, 0.90)	0.23 (-0.56, 0.80)	0.58 (-0.20, 0.91)	0.45 (-0.36, 0.87)	0.47 (-0.34, 0.88)	0.6 (-0.17, 0.91)	-0.61 (-0.92, 0.15)
**3**	0.75 (0.00, 0.96)	-0.04 (-0.77, 0.73)	0.55 (-0.34, 0.92)	0.53 (-0.36, 0.91)	0.41 (-0.49, 0.89)	0.30 (-0.58, 0.86)	0.71 (-0.08, 0.95)	-0.42 (-0.89, 0.48)

CI= confidence intervals

© 2024 Shukla VV. *JAMA Network Open.*

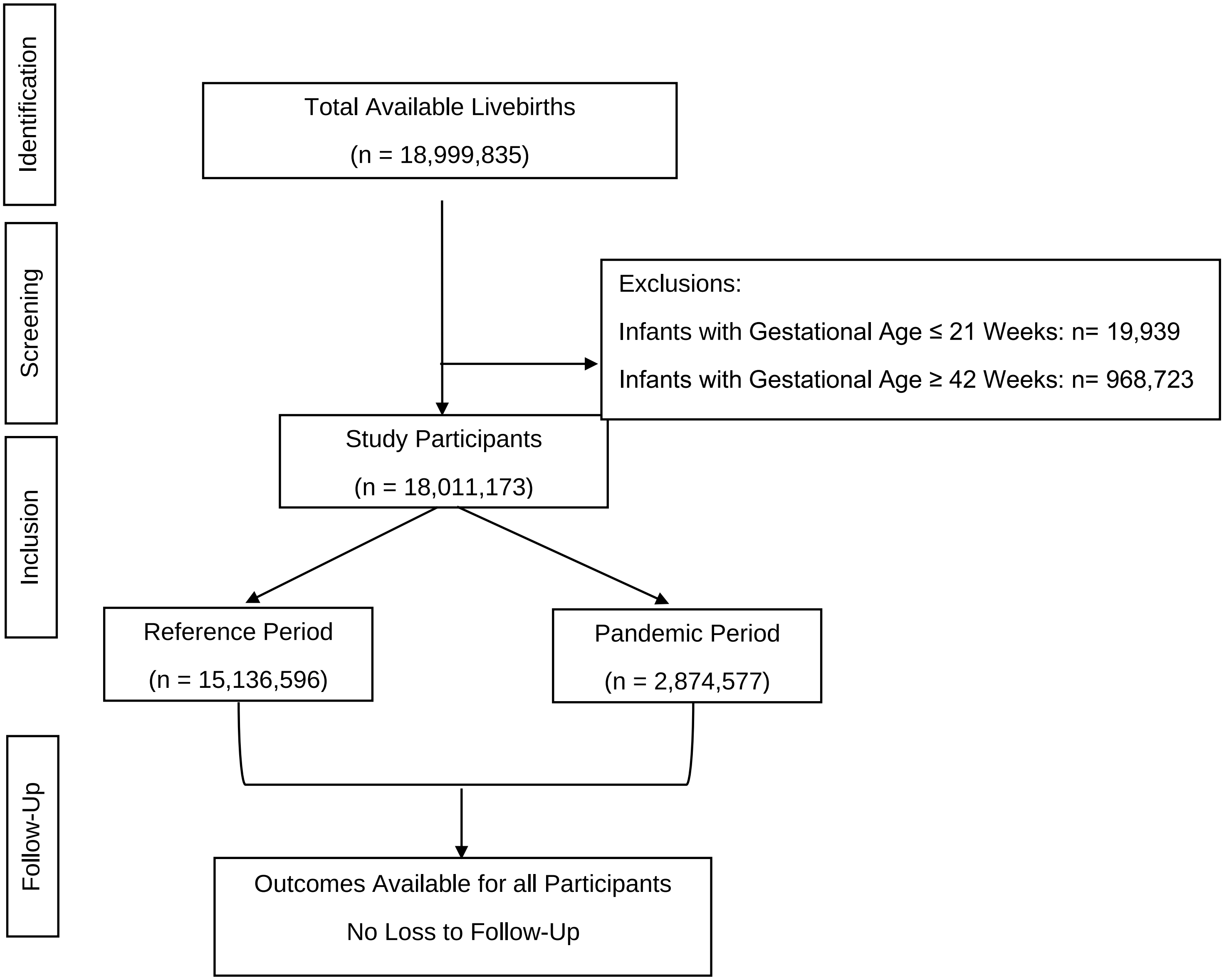

eFigure 1: Participant Flow Diagram.

© 2024 Shukla VV. *JAMA Network Open.*

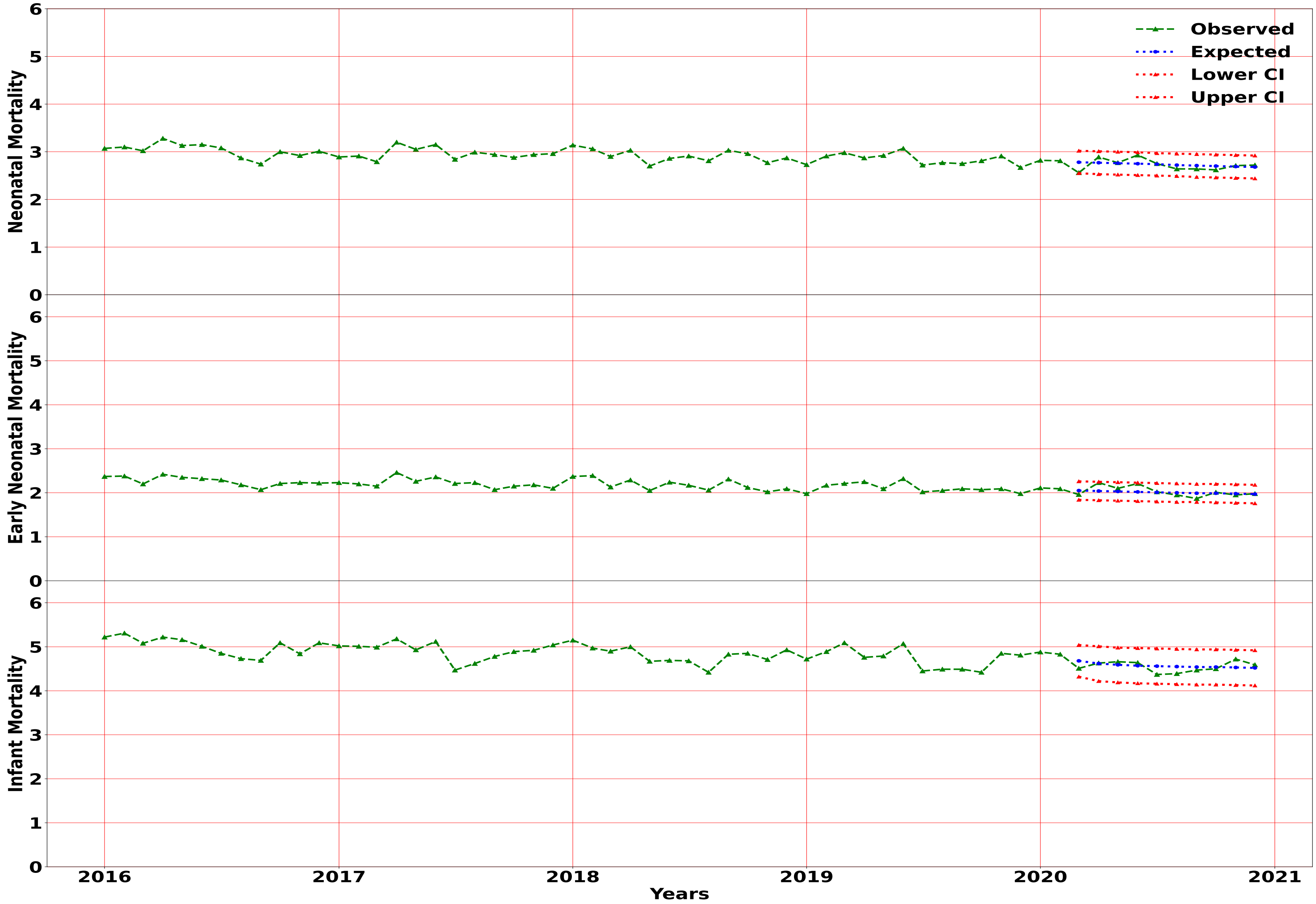

eFigure 2: Study Period Observed and ARIMA Expected Pandemic Period Neonatal, Early Neonatal, and Infant Mortality Rates. Rates per 1,000 live births**.** All observed mortality rates fell within the upper and lower confidence intervals of the ARIMA model expected rates. CI= confidence intervals.

© 2024 Shukla VV. *JAMA Network Open.*

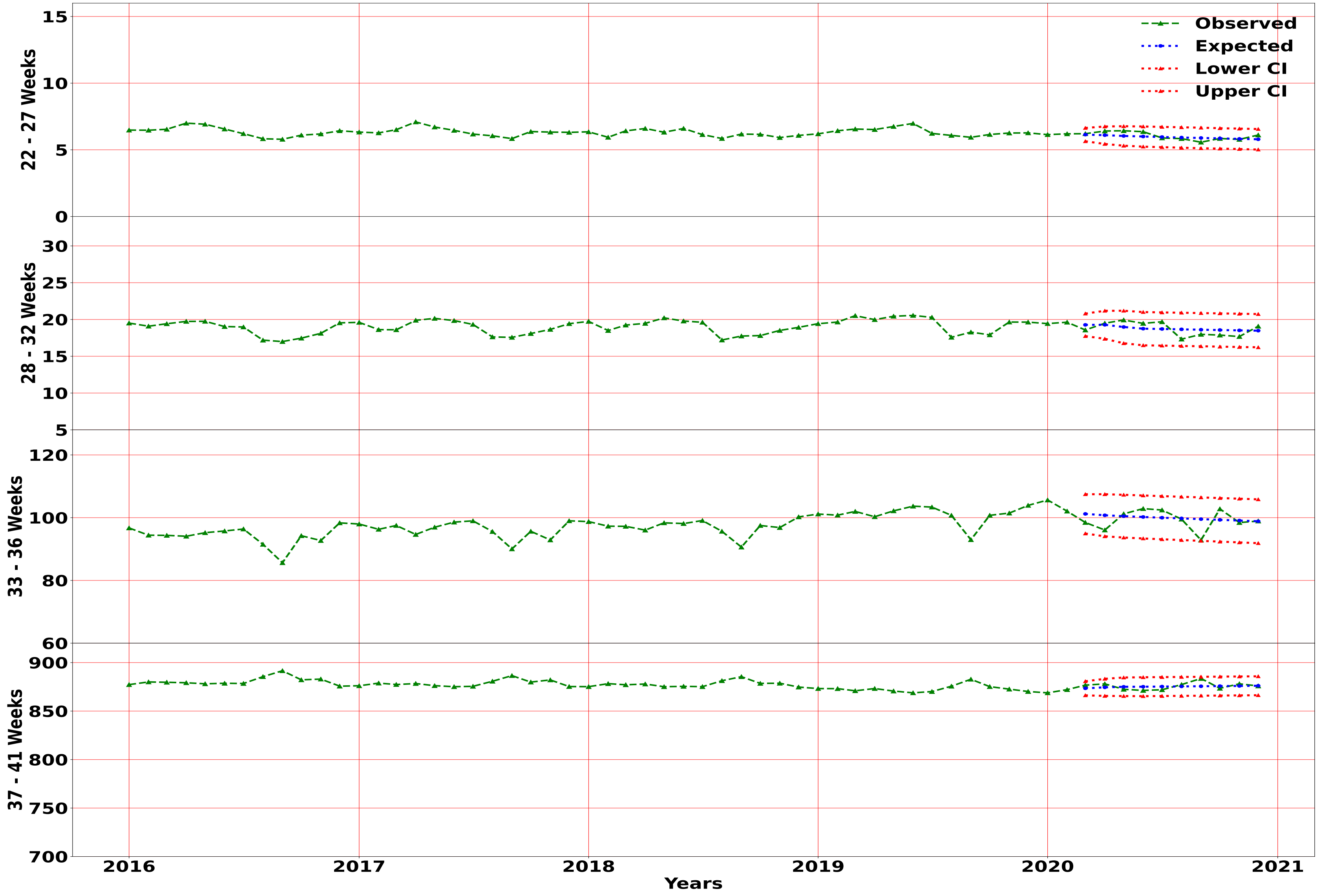

eFigure 3: Study Period Observed and ARIMA Expected Pandemic Period Births at Gestational Age of 22-27 Weeks, 28-32 Weeks, 33-36 Weeks, and 37-41 Weeks. Rates per 1,000 live births**.** All observed gestational age at birth rates fell within the upper and lower confidence intervals of the ARIMA model expected rates. CI= confidence intervals.

© 2024 Shukla VV. *JAMA Network Open.*
