# Supplementary material for: Social Distancing During the COVID-19 Pandemic and Neonatal Mortality in the US
Source: JAMA Netw Open. 2024 Jul 18;7(7):e2422995. doi: 10.1001/jamanetworkopen.2024.22995 (PMC11258585; doi:10.1001/jamanetworkopen.2024.22995)
Supplement: Supplement 2. — Data Sharing Statement [file jamanetwopen-e2422995-s002.pdf]

Data Sharing Statement

Shukla. Social Distancing During the COVID-19 Pandemic and Neonatal Mortality in the US. *JAMA Netw Open*. Published July 18, 2024. doi:10.1001/jamanetworkopen.2024.22995

Data
Data available: No

Additional Information
Explanation for why data not available: The data for the study was from the the National Center for Health Statistics of the Centers for Disease Control and Prevention maternal linked birth and infant death records. Access to this data and data dictionary has to be requested directly.
